# Benign Nevi Mimicking Melanoma: A Diagnostic Dilemma

**DOI:** 10.7759/cureus.74821

**Published:** 2024-11-30

**Authors:** Aisha Tabassum, Mohammad S Iqbal

**Affiliations:** 1 Medicine, Batterjee Medical College, Jeddah, SAU

**Keywords:** benign nevi, clinic-pathological correlation, congenital nevus, dysplastic nevus, halo nevus, immunohistochemistry mimics, melanoma, spitz nevus

## Abstract

The incidence of melanoma is increasing worldwide and is not restricted as previously to fair-skinned individuals and one of the main contributing factors is the drastic development of diagnostic approach to the melanocytic lesions. Additionally, melanomas are often misdiagnosed (underdiagnosed and overdiagnosed) as many benign melanocytic lesions such as Spitz nevus, deep penetrating nevus (DPN), compound nevus, and regenerating nevi exhibit some features of melanoma. Clinico-pathological correlation is of utmost importance in the diagnosis of such lesions. Cytological details should be carefully studied in addition to a good “low power” assessment of the growth pattern. Appropriate immunohistochemistry (IHC) is necessary whenever needed as misdiagnosis has deleterious consequences for both the patient and the pathologist.

## Introduction and background

Cutaneous melanocytic lesions are one of the most common tumors especially among Caucasians with fair skin [1]. The advent of a developed diagnostic approach has led to early detection of melanoma and hence, contributed to the increased incidence of diagnosis [2]. Most benign lesions are easily diagnosed clinically, as well as pathologically, due to the presence of established diagnostic criteria [1]. However, very often benign nevi are misdiagnosed as melanoma due to some overlapping features, unusual sites, and partial biopsies [2-4]. Some lesions show atypical features and are difficult to diagnose such as dysplastic nevi (DN), the most common mimicker of melanoma. Some nevi are potential precursors of melanoma such as DN and to a lesser extent, common acquired nevi and congenital nevi. However, most of these lesions are stable and will not progress to malignancy, and hence, complete excision is not compulsory in these cases. Special sites such as breast, flexural skin, acral region, and genitalia may show atypical features suggestive of melanoma. However, these are not premalignant, and overdiagnoses may lead to excessive treatment for the patient. Conversely, underdiagnosis of melanoma can lead to late diagnosis and loss of curable stage for the patient [5]. In this review, we shall discuss the potential benign melanocytic mimics of melanoma.

## Review

General consideration

The accuracy of histopathology reports in melanocytic lesions is partly dependent on the relevant clinical history provided in addition to other factors such as the amount of tissue provided and the type of biopsy done [4]. The ABCD rule was first introduced in 1985 and then expanded in 2004 to the ABCDE rule, which still holds good in clinically differentiating melanomas from benign melanocytic lesions. These features include asymmetry, borders that are irregular, color variegation within the lesion as well as from the patient’s other nevi, a diameter greater than 6 mm, and an evolving lesion that is either a new or changing lesion [6]. The term “ugly duckling” was later added to this list, which indicates a spot that is unlike the other nevi and is more likely suspicious of malignancy. These criteria provide an easy framework to correlate the histopathological findings with those of clinical features [7].

Nevi are far more common than melanomas and their growth is very slow [5]. Age, gender, and site of the lesion are equally important. Nevi occurring on certain locations such as acral regions, scalp, breast, genital skin, ear, and flexural sites can exhibit atypical features such as asymmetry, focal pagetoid growth, and or single cell growth, which are generally seen in melanomas. Interpretation of mitotic activity will be based on the age of the patient. Congenital nevi and Spitz nevi in young children might show increased activity, whereas, in adults, it mainly signifies melanomas [4,8]. Melanomas with Spitzoid features are often seen in young males but after 35 years of age, a reverse trend is seen. Benign nevi in pregnancy shows an increased mitotic rate [3].

Microscopically at “low-power view,” the benign lesions are characterized by symmetry, circumscription, superficial distribution, and smaller size. However, there are exceptions to all these criteria. For example, melanomas can be of smaller size, but mimics of melanoma, such as Spitz nevus and Reed’s spindle cell nevus, should always be considered before diagnosing small lesions as melanomas. Congenital nevi, deep penetrating nevi (DPN), cellular blue nevi (CBN), and some Spitz nevi may extend deeply into the dermis; However, lesions that do not fulfill the criteria for these entities and extend into the deeper dermis should always be suspected as melanoma [8]. Regressing tumors (benign and malignant) may show a cellular response in the form of lymphocytic infiltrate. Periretal lamellar collagen is a feature of DN [3]. The characteristic features differentiating benign nevi from melanoma are shown in Table 1.

**Table 1:** Characteristic features of benign nevi and melanoma

Features favoring benign nevus	Features favoring melanoma
Younger age	Older age group
Symmetric	Asymmetric
Well-circumscribed	Irregular borders
Diameter <6 mm	>6 mm in diameter
Uniform color	Variegated appearance
No pagetoid spread within epidermis	Pagetoid spread in epidermis
Uniform cellularity	Cytological atypia
Deep maturation	Lack of maturation
Deep mitoses generally absent	Deep mitoses present
No vascular invasion and neurotropism	Vascular invasion, neurotropism, and satellites

The most common melanocytic nevi that mimic melanoma and the practical problem with partial biopsies are included in Table 2. Each entity will be described with emphasis on features overlapping with melanoma and important points to differentiate the two from one another.

**Table 2:** Common melanocytic nevi that mimic melanoma and partial biopsies that hinder diagnosis

1	Congenital nevus
2	Dysplastic nevus
3	Desmoplastic nevus
4	Recurrent/traumatized nevus
5	Spitz nevus
6	Atypical Spitz tumor
7	Spindle cell nevus
8	Halo nevus
9	Deep penetrating nevus
10	Cellular blue nevus
11	Combined nevus
12	Nevus in pregnancy
13	Acral nevus
14	Nevi of special site
15	Partial biopsies

Congenital nevus

Giant congenital melanocytic nevus (GCMN) is a great mimicker of malignant melanoma. Changes in color, size, shape, rapid growth rate, nodularity, and even ulceration, which are very often seen in melanoma, may occur in this benign lesion [9].

Histopathological clues to a benign lesion include small, monotonous nuclei; if atypia is present, it is limited to the epidermis and superficial dermis. The atypical junctional component does not extend laterally beyond the intradermal component, and the pagetoid extension tends to overlap nests [8].

Benign proliferative nodules (PN) in GCMN may mimic melanoma, both clinically and histologically. However, PNs are usually present at birth as multiple nodules, which are not seen in cases of melanomas, and despite their alarming appearance, with time they reduce in size, become softer, and may even completely disappear. Microscopically, the typical features of malignancy such as pleomorphism, mitosis, and pagetoid spread are absent, and even if present, they regress with time [10,11].

Neonatal malignant melanoma is extremely rare, arising as malignant transformation in GCMN or as metastases from the mother [11]. Most metastasizing melanomas arising in congenital nevi in children show atypical nuclei mainly in the deep dermis or subcutis. However, in the case of adults, a melanoma arising in a congenital nevus will typically have a conventional in situ component. Thus, an atypical junctional melanocytic proliferation within a congenital nevus in an adult is suspicious [8].

Dysplastic nevus

DN are great mimickers of melanoma both clinically and histologically. Clinically, they are characterized by four major properties: their size tends to be greater than that of common nevi but less than that of melanomas (>5 mm), macular/flat lesions with a pebbly surface, fuzzy/indistinct borders in contrast to superficial spreading melanoma, and variegated appearance. Hence, they overlap the ABCDE criteria for the diagnosis of melanoma [5].

DN are characterized by lentiginous hyperplasia, perirectal lamellar collagen, architectural disorder, and cytological atypia. The cytological atypia may be mild, moderate, or severe [2,3]. Often the atypia is random as opposed to diffuse atypia in melanoma [8]. DN becomes concerning when there is moderate to severe atypia, focal infiltration of the epidermis, and dermal mitoses. However, these are not problematic individually; only in combination, or when associated with asymmetry, poor maturation, and an expansile dermal component, do they raise the possibility of melanoma [1].

Facial lesions, especially in the context of solar elastosis, should be carefully evaluated for dysplasia, as these features overlap with those of lentigo maligna melanoma. All such lesions should be completely excised with a margin of normal tissue. Finally, melanoma can be seen in association with a DN and will show characteristic features, such as asymmetry of the junctional component and any associated lymphocytic infiltrate, confluence of junctional nests, and widespread pagetoid spread. All such lesions should be completely excised [8].

Desmoplastic nevus

Differentiation of desmoplastic melanoma (DM) from desmoplastic nevus is extremely difficult in small superficial biopsies and inadequate clinical history. Features that favor melanoma over nevus include the large size, sun-exposed area, infiltrative growth pattern, asymmetry, and numerous cells with atypical nuclei. Morphologically cells resemble fibroblasts in a scar; however, the overlying epidermis might show increased and enlarged melanocytes representing the in-situ component. Scattered mitotic figures are seen in the dermis [12,13]. Nerve infiltration is often seen, especially in association with head and neck tumors. The extent of nerve involvement may vary from focal perineural invasion to extensive neurotropism [14]. Lymphocytic infiltration of the dermal component is a feature of DM rather than desmoplastic nevus [8].

DNs on the other hand are generally smaller lesions with a wedge-shaped silhouette, with a diminution in cellularity toward the deeper aspects of the lesion. The cells are larger, either epithelioid or spindle-shaped, with abundant cytoplasm and clear cytoplasmic borders [8]. Atypia, mitoses, or lymphocytic foci are generally absent [3].

Recurrent and traumatized nevus

Recurrent nevi are seen characteristically in young women, most often on the back, followed by the face and extremities with recurrence appearing within six months of the original biopsy [15,16]. It usually presents as a macular area of scar with variegated pigmentation, linear streaking, or even diffuse pigmentation patterns. The original nevi undergoing recurrence are often the commonly acquired nevi and less commonly other nevi such as congenital, dysplastic, and Spitz nevi, and rarely specialized nevi such as blue and spindle cell nevi [16].

Recurrent nevi are characterized by the classical tri-layered appearance of the lesion wherein the atypical proliferation overlies an area of granulation tissue or scarring in the upper dermis, and the residual banal nevus cells are seen beneath the granulation tissue. However, atypical proliferation is usually confined to the area of trauma and appears well-circumscribed [8].

RN is often called pseudo-melanoma as it can present with atypical features such as irregularity, asymmetry, poor circumscription, cytological atypia of the junctional component, and pagetoid spread mainly in the center of the specimen. The dermal component is usually bland and shows maturation. In all such cases, history of previous surgery, presence of residual nevus cells, and dermal scarring of the previous nevus should always be searched [17].

It is clinically difficult to differentiate a recurrent nevus from a recurrent melanoma. Incomplete removals of these lesions by shave or punch biopsy or even after trauma results in the recurrence of pigmentation. In all such cases, it is often difficult or impossible to get the original pathology report or slides [18].

Spitz nevus

Spitz nevi show a range of histological features, from benign to malignant lesions, such as Spitz tumor, atypical Spitz tumor (AST), and malignant melanoma [19].

Spitz nevus is often seen in younger individuals less than 20 years old, often on the face and lower extremities, presenting as a solitary, small papule or nodule less than 1 cm in size. These lesions are often symmetric, well-defined, red to tan in color, and grow rapidly over a period of months [2]. Classic Spitz nevi can be compound (most common), junctional, or intradermal [2,20].

Spitz nevi are composed of epithelioid to spindled melanocytes with abundant homogenous cytoplasm and large vesicular nuclei, and often display epidermal hyperplasia, clefts around and between melanocytes, Kamino bodies, and maturation with descent. They can have limited scatter of melanocytes into the upper levels of the epidermis [21]. Dermal nevus cells are nested in the superficial part and show maturation with a reduction in cell size and dispersion of nests by collagen bundles in the deeper dermis. Dermal architecture is always maintained. Mitotic activity is absent and even if present, is usually sparse, and is limited to the junctional and superficial dermal components. Dusty pigmentation is occasionally seen, and mostly the lesion lacks pigmentation [20].

The lesion becomes suspicious for melanoma in cases with an age of more than 40 years, a diameter of >1 cm, asymmetry, ulceration, and poor circumscription.An epidermal component with an extensive pagetoid pattern extending laterally, a dermal component lacking maturation or extending into the subcutaneous tissue, or with a high mitotic count, marked cytological atypia, and brisk lymphocytic infiltrate in the lesion also points toward malignancy [22].

Atypical Spitz tumor

These are the lesions that do not fall in the category of typical SN but also do not fulfill the required criteria for melanoma [23]. Atypical Spitz nevi are conventional Spitz nevi with one or more atypical features such as larger size (>6 mm), irregular borders, and ulcerated lesions. Microscopically, asymmetry, ulceration, pagetoid melanocytosis, lack of maturation in deeper parts, deep mitoses, and cytological atypia can be seen. There are no clear histological criteria that differentiate atypical Spitz nevi from malignant melanoma [19]. Some authors refer to them as ASTs of uncertain malignant potential and recommend complete excision of the lesion with sentinel lymph node biopsy for lesions >1 mm in thickness to rule out malignant potential [1].

Pigmented spindle cell nevus of Reed

The pigmented spindle cell nevus (PSCN) of Reed was once considered a pigmented variant of a spindle cell type Spitz nevus. It may mimic melanoma both clinically and in histopathology. The most common clinical presentation is a small, relatively well-circumscribed, symmetrical plaque-like papule with smooth discrete borders and a darkly pigmented lesion. They often appear suddenly but remain stable till they are excised, as seen on the thigh or legs of young adult females [24].

Features that overlap with Spitz nevus include symmetry, diffuse epidermal thickening, and hypergranulosis. However, PSCN is often heavily pigmented compared to Spitz nevus. Kamino bodies and epidermal spread may also be seen creating difficulty in differentiating it from melanoma. The dermal component is often small and shows maturation. The clinical correlation as well as architectural and cytological features help in the final diagnosis in such cases [1].

Halo nevus

Halo nevus is also known as Sutton’s nevus where the nevus is surrounded by a halo of depigmentation more commonly seen on the back. These are benign nevi being symmetrical round to oval, often multiple lesions, affecting young children and teens, seen in association with a family history of vitiligo. Depigmentation is a T-cell-mediated immune response to the antigens of the nevus cells [25].

Microscopically, the halo phenomenon refers to the dense lymphocytic infiltrate around the centrally placed nevus cells, surrounding or infiltrating the nevus cells, which can be a congenital melanocytic nevus, a blue nevus (BN), Spitz nevus, or an atypical nevus [26]. These lymphocytes can obscure the nests at the dermo-epidermal junction and the dermis [27].

Halo nevus is often easily diagnosed on clinical examination; however, on histopathology, it can mimic melanoma with regression. Halo nevi show atypical cells only at the junctional nests and upper portion of the nevus, with maturation toward the base [26,27]. There is no epidermal invasion, and atypia is mild to moderate [1]. Inflammatory infiltration is often distributed in a band-like pattern, symmetrically around the nevus cells, whereas in regressing melanomas, the distribution is asymmetric, surrounding only some segments of the tumor [1,26]. Occasional mitoses and younger age groups of the patient favor a benign nevus [27].

Deep penetrating nevus

Conventional DPN is a benign nevus, seen in young females in their second and third decades, often presenting as solitary lesions <1 cm in size, appearing as a symmetric papule, macule, or nodule - non-ulcerated, dome-shaped, and darkly pigmented - located in the head and neck region [28-30].

Histologically, DPNs are small, well-circumscribed, symmetrical lesions consisting of spindle and/or epithelioid melanocytes, and occasionally fusiform cells, arranged in fascicles, cords, and nests involving the deep dermis and subcutaneous tissue in a wedge-shaped manner. The melanocytes may run along in association with neurovascular and adnexal structures. A Grenz zone and collagen trapping might also be seen. The presence of sparse to abundant heavily pigmented melanophages is characteristic. There may be a limited junctional component and lymphocytic infiltrate [28,29,31]. Cells are large but cytological atypia and mitoses are rare and DPNs have an indolent behavior [27,31]. Maturation is generally absent. DPNs can be seen in association with other nevi, such as Spitz nevus, conventional nevus, and BN, as a compound nevus [28].

DPN can be differentiated from melanoma as primary cutaneous melanoma is an asymmetrical and poorly circumscribed proliferative lesion, with a definite junctional component of lentiginous or pagetoid proliferation composed of atypical melanocytes with destructive growth in the dermis. Melanomas are continuous lesions, and the DPNs can show discontinuity of the lesion in the papillary dermis. The dermal component in melanomas is in the form of expansile nests or sheaths of atypical melanocytes. Cytological atypia in melanomas is more marked than in DPN with frequent mitoses [28,29].

Cellular blue nevus

Blue nevi (BN) are a heterogeneous group of congenital and acquired melanocytic lesions that include common BN/dendritic blue nevus (DBN), cellular blue nevus (CBN), atypical cellular blue nevus (ACBN), and melanoma in BN [32]. They are derived from the melanocytes of neural crest origin that become arrested during the embryonic process of migration to the epidermis, and they remain within the dermis. An aggregation of such dendritic melanocytes as a mass in the dermis is called the BN [33].

DBN and CBN are microscopically characterized by pigmented dendritic and spindled melanocytes. These lesions usually present as blue, gray, or black solitary nodules, on extremities in case of the common BN or sacro-coccygeal and gluteal region in case of cellular variant in young females [34,35]. The blue color of the lesion is due to the Tyndall phenomenon, where the lesion absorbs the longer, less energetic wavelengths of visible light into the dermis [33].

Microscopically, DPNs are wedge-shaped lesions composed of spindled, dendritic melanocytes proliferating in the dermis, with elongated, hyperchromatic nuclei and variable amounts of pigmentation. There is an associated desmoplastic stromal reaction and melanophages. CBNs typically grow as larger, multilobulated masses with a dumbbell-shaped appearance, composed of a biphasic proliferation of dendritic melanocytes, like those seen in DBNs, admixed with cellular nodules of spindle- or oval-shaped melanocytes and a variable admixture of pigmented macrophages [34].

Malignant BN usually has a multinodular appearance, with progressive growth, generally larger in size (more than 3 cm in diameter), and located on the scalp. It can arise from a BN, nevus of Ota or Ito or de novo, and its biological behavior is uncertain [32]. Histological evidence of malignant transformation within a BN includes loss of the normal biphasic architecture with the development of a sheet-like growth pattern, variable necrosis, nuclear hyperchromasia and pleomorphism, prominent nucleoli, epithelioid morphology, excessive mitotic activity, and an infiltrative border [35,36]. These atypical cytological features are not directly present adjacent to bland-appearing nevus cells making the diagnosis even more difficult [36]. "Some authors have suggested that the most important criterion for distinguishing a benign lesion from its malignant counterpart is the presence of widespread necrosis [35,36].

Combined nevus

Any nevus composed of two morphologically different nevus cells is called a combined nevus. The two components may be separate or intermingled, in which case they may have a heterogeneous appearance and may appear asymmetric mimicking melanomas [1]. These lesions can occur in any age group, being more common among females [37]. The most common combinations include a conventional nevus and a BN, characterized by a population of dendritic melanocytes, which are often heavily pigmented with prominent melanophages, mixed with the more conventional nevoid cells. The BN component might show mitoses in their dermal component [8]. Other combinations more frequently seen include Spitz or PSCN combined with another type of nevus such as common nevus or DN, a DPN with a common nevus, and other combinations including two or more nevus types. Many of these combinations show atypical features making the diagnosis difficult [37].

Despite their heterogeneous appearance or asymmetry, the combined nevi lack clear-cut features of malignancy such as poor maturation, in situ component, nuclear atypia, frequent dermal mitoses, and lymphocytic infiltrate [1]. Sheet-like growth pattern, infiltrative cords of pleomorphic cells, regressive fibrosis, desmoplastic reaction, pagetoid intradermal infiltration by markedly atypical melanocytes, and extensive mitoses always favor melanoma over a combined nevus [1,8,37].

Nevus in pregnancy

Pre-existing benign nevi such as common nevus and BN might rapidly grow during pregnancy, acquire irregular borders and pigmentation, and show junctional activity [1,38]. Dermal components show increased mitotic activity throughout the lesion; however, the mitosis is typical with a lack of nuclear pleomorphism and nucleoli [1]. All such lesions should be interpreted carefully keeping in mind that the melanomas in pregnancy are very aggressive tumors [39].

Acral nevus

Nevi arising on palm, soles, and nail beds (non-hair bearing sites) are called the acral nevi [2,40]. They are generally small lesions but have unusual gross and microscopic features not seen in other benign nevi [2]. Acral nevi are more common among Asians and in dark-skinned people. Microscopically, these are junctional or compound melanocytic nevi, which are symmetric and well-circumscribed. One characteristic feature includes lentiginous and nested junctional melanocytic proliferation. These nests differ in size and are often vertically oriented. Pagetoid scatter, bridging between rete ridges and fibroplasia, is usually confined to the center of the lesion. Dermal components, if present, show maturation and lack of mitotic activity with mild cytological atypia [4,40].

Nevi of special site

Nevi of certain sites in the body such as genitalia, ears, scalp, breast, flexural skin, shoulder, legs, ankle, and back exhibit microscopic features that mimic melanoma such as asymmetry, irregular borders, pagetoid spread, cytological atypia, and mitoses. A careful interpretation must be done at all these sites to avoid an overdiagnosis of melanoma [5,41]. Features favoring benign nevus include small size, nested growth pattern, younger age group, and a bland intradermal component. Alternatively, features such as a predominantly lentiginous growth pattern, a junctional component in the elderly, and a dermal inflammatory infiltrate favor melanoma [8].

Partial (shave/punch) biopsies 

An excisional biopsy specimen is ideal for assessment of melanoma. Other specimens, such as punch biopsy, shave, and curettage, may become distorted and may not allow adequate assessment of the important parameters needed to differentiate benign lesions from melanoma, such as symmetry, architecture, and focally suspicious changes, as well as Breslow’s thickness, mitotic rate, and other factors required for definitive treatment [3,4]. These partial specimens are not representative of the lesion and usually lack the edges and bases of the lesions, which are vital in assessing the prognosis of melanoma [3]. In case of a discrepancy between clinical and pathological diagnosis, multidisciplinary meetings should be arranged and the case can be referred to another pathologist with expertise and skill in melanocytic lesions [3,4]. Once the diagnosis of melanoma is established, wide re-excision is a must to ensure that the whole lesion is removed, in order to reduce the risk of local recurrence. Whenever a wide re-excision specimen is submitted, the pathologist should know whether the melanoma was originally reported as completely excised, whether the initial biopsy specimen was a punch, shave, or excision, and whether the margins (deep and peripheral) in the case of a shave biopsy were free or involved [4]. The atypical features seen in benign nevi that overlap with the features seen in melanoma are depicted in Table 3.

**Table 3:** Benign nevi showing features overlapping with melanoma GCMN: giant cell melanocytic nevus, PN: proliferative nodule, DN: dysplastic nevus, RN: recurrent nevus, DPN: deep penetrating nevus, AST: atypical Spitz tumor

Features favoring melanoma	Benign nevus showing feature overlapping with melanoma
Asymmetric	GCMN, DN, RN, AST
Irregular borders	GCMN, DN, RN
>6 mm in diameter	GCMN, DN, AST
Variegated appearance	GCMN, DN, RN
Evolving lesion	
Pagetoid spread in the epidermis	PN in GCMN, RN, AST
Cytological atypia	PN in GCMN, DN, RN, AST
Lack of maturation	AST, DPN
Deep mitoses present	PN in GCMN, AST

Pigmented epithelioid melanocytoma

Pigmented epithelioid melanocytoma (PEM) was first described as animal-type melanoma due to its resemblance to tumors occurring in horses, which are often heavily pigmented; However, presently it is categorized as intermediate grade or borderline melanocytic neoplasm [42-44]. It is characterized by its indolent behavior, occurring in young individuals with equal sex predilection [45]. PEM can occur as sporadic cases, as well as part of Carney’s syndrome, showing loss of expression of cyclic AMP-dependent protein kinase 1R1a in the latter case. This protein is involved in the regulation of melanocytic proliferation and pigment production. Therefore, the loss of R1a expression in PEM explains the dark pigmentation of this tumor [46]. Microscopically, PEM tumors have sheets and nodules of heavily pigmented melanocytes in the deep dermis consisting of a dual population of cells with epithelioid and spindled appearance. Epithelioid cells are often in the center of the lesion, whereas spindle cells are seen in the periphery often distributed as single cells or small groups. Melanophages form a small population of about 10% of the lesion. PEM often extends deeply into the dermis and subcutaneous tissue along periadnexal tissue. PEM shows low mitotic activity, lack of regression, or vascular invasion [44].

Immunohistochemistry

Histopathology is still the gold standard for the diagnosis of melanoma and benign melanocytic lesions. Applications of immunohistochemistry (IHC) in difficult melanocytic lesions are often unhelpful, can waste the tissue, and also require considerable experience. No single marker or even a panel can give an unequivocal diagnosis in these difficult lesions [8,47]. Analysis of the pattern of staining (patchy vs. diffuse), maturational gradient, and correlation with morphological features should be considered for proper interpretation. Certain lesions, e.g., DM and BN, have their own characteristic IHC fingerprints. IHC data must be always interpreted and correlated with clinical and histopathologic findings [48].

Ki-67, HMB45, and p16 are some of the markers helpful in differentiating a nevus from a melanoma. Ki-67 index is expected to be higher in melanoma than in benign nevi. A strong Ki-67 index favors melanoma but does not eliminate melanoma as some melanomas can have low proliferation [48].

HMB-45 labels primarily intra-epidermal melanocytes, activated melanocytes, and immature melanocytes [47]. In the case of benign nevi, the staining is mainly seen in the epidermis and the upper portion of the lesion and/or around the adnexa, whereas the deep dermal component is generally negative [8,47,49]. This gradient of staining is lost in cases of melanoma. BN, Spitz nevi, and DPN are exceptions and can show diffuse staining throughout the lesion [8,47,48]. Additionally, the gradient pattern of staining with HMB-45 is not useful in melanoma in situ cases [50].

p16 IHC shows loss of staining in the case of melanoma due to biallelic loss of chromosome 9p21, a molecular aberration seen in melanoma, whereas strong patchwork p16 staining throughout the lesion favors a benign lesion. However, negative staining could be interpreted with caution, and technical errors need to be ruled out [8].

MIB-1 can be used to assess the proliferative activity in certain lesions such as Spitzoid tumors, and a combination of Melan-A will ensure the origin of the lesion as melanocytic [8].

Mart-1/Melan-A is a cytoplasmic protein expressed in adult resting melanocytes and melanomas. It can label lentiginous proliferation and pagetoid spread of melanocytes, highlighting their extent, which is useful in distinguishing DN from melanoma [51].

Preferentially expressed antigen in melanoma (PRAME) is a tumor-associated antigen isolated by autologous T-cells. This recently developed IHC marker is overexpressed in melanoma rather than benign melanocytic lesions [50,52]. As for melanocytic nevi, they are generally negative or express very low levels of PRAME [52,53]. Focal PRAME positivity is seen in commonly acquired nevi, as well as dysplastic, traumatized, and recurrent nevi. PRAME positivity does not equate to malignancy [52].

5-hydroxymethylcytosine (5-hmC) is an epigenetic marker that is lost in melanoma and is emerging as a useful IHC marker for differentiating atypical benign nevi from melanomas [8,54,55]. 5-hmC staining is intense and 100% in normal basal layer melanocytes, whereas commonly acquired nevi and low-grade DN show 60% of melanocytes staining intensely. High-grade DN displays 90% lightly stained to 10% negatively stained melanocytes, and staining is nearly completely lost in melanoma [54].

## Conclusions

In the case of difficult melanocytic lesions, complete excision of the lesion is mandatory. Overdiagnosis and underdiagnosis should be avoided to prevent unnecessary treatment and anxiety for the patient, and a second opinion should always be sought. Good communication with clinicians and the application of standard criteria for diagnosis, along with the appropriate use of IHC, can go a long way in providing patients with an accurate diagnosis and treatment. All atypical nevi should be monitored for early detection of melanoma.
